# Adamantinomatous craniopharyngioma as a model to understand paracrine and senescence-induced tumourigenesis

**DOI:** 10.1007/s00018-021-03798-7

**Published:** 2021-03-26

**Authors:** Jose Mario Gonzalez-Meljem, Juan Pedro Martinez-Barbera

**Affiliations:** 1 Tecnologico de Monterrey, School of Engineering and Sciences, Mexico City, Mexico; 2 Developmental Biology and Cancer Research and Teaching Programme, Birth Defects Research Centre, UCL Great Ormond Street Institute of Child Health, London, UK

**Keywords:** Pituitary tumour, Cancer stem cells, SOX2, WNT/β-catenin, Oncogene-induced senescence, Therapy-induced senescence, Senolytics

## Abstract

Cellular senescence is a process that can prevent tumour development in a cell autonomous manner by imposing a stable cell cycle arrest after oncogene activation. Paradoxically, senescence can also promote tumour growth cell non-autonomously by creating a permissive tumour microenvironment that fuels tumour initiation, progression to malignancy and metastasis. In a pituitary tumour known as adamantinomatous craniopharyngioma (ACP), cells that carry oncogenic β-catenin mutations and overactivate the WNT signalling pathway form cell clusters that become senescent and activate a senescence-associated secretory phenotype (SASP). Research in mouse models of ACP has provided insights into the function of the senescent cell clusters and revealed a critical role for SASP-mediated activities in paracrine tumour initiation. In this review, we first discuss this research on ACP and subsequently explore the theme of paracrine tumourigenesis in other tumour models available in the literature. Evidence is accumulating supporting the notion that paracrine signalling brought about by senescent cells may underlie tumourigenesis across different tumours and cancer models.

## Introduction

Almost over 60 years ago, it was first reported that continuous in vitro culturing of human cells results in a gradual but ultimately complete decay of their proliferative capacity [1, 2]. The term *cellular senescence* was then applied to describe this particular phenomenon as it was hypothesized to be the result of a deterioration in the cell’s homeostatic functions with time, a process resembling organismal aging [3]. However, recently acquired understanding of the complexity and heterogeneity of this phenomenon has revealed that senescent cells can be anything but a simple manifestation of decay and dysfunction, as their name might otherwise suggest.

The early concept of cellular senescence has now been expanded to describe a growing list of phenotypes initiated by damaging stimuli such as telomere attrition, ionizing radiation, chemotherapeutic compounds, reactive oxygen species (ROS), mitochondrial dysfunction and oncogenic signalling [4]. Importantly, all of these phenotypes share common hallmark features such as the activation of DNA-damage pathways, cell cycle arrest mediated by the p16^INK4^/Rb and p21^CIP1^/p53 pathways, the activation of anti-apoptotic mechanisms and the widespread secretion of growth factors, cytokines, chemokines and extracellular matrix components (collectively known as the senescence-associated secretory phenotype or SASP). The different types of senescent phenotypes and their underlying mechanisms have been thoroughly reviewed elsewhere [4, 5].

Senescent cells and the SASP can induce a vast array of context-dependent effects, playing significant roles in the regulation of normal tissue physiology but also in disease. Senescent cells can be found in several tissues during embryonic development and participate in the proper patterning of some organs and tissues [6–9]. After development, senescent cells are also involved in tissue regeneration and wound repair in several organs, although their exact role appears to be more complex and context dependent. While they have been reported to play beneficial roles in acute wound repair [10–16], the opposite has been observed during chronic wounding scenarios [17–20]. This detrimental aspect of long-term senescent cell accumulation has also been widely described in the development of several pathologies, including those related to organismal ageing (e.g. atherosclerosis, rheumatoid arthritis, metabolic dysfunction, diabetes and neurodegenerative diseases, among many others). It is possible that this dichotomy is related to a tight regulation of dynamic balances between contrasting SASP activities, such as the paracrine promotion of cellular plasticity and reprogramming on one side, and the induction of by-stander senescence and inflammation on the other [21, 22]. Importantly, there is evidence demonstrating that the SASP can lead to widespread effects beyond the microenvironment, such as driving systemic inflammation and haemostasis, as well as mediating several side effects of chemotherapy including decreased physical activity and strength, bone marrow suppression and cancer recurrence [23–26]. Both detrimental and beneficial activities of senescent cells and the SASP have previously been reviewed in detail [27–29].

In the case of cancer and neoplastic diseases, senescence can be induced cell autonomously by oncogene activation (i.e. oncogene-induced senescence, OIS) or through therapeutics such as DNA-damaging chemical compounds and ionizing radiation (i.e. therapy-induced senescence, TIS), which lead to the activation of DNA-damage pathways and the activation of a stable cell cycle arrest [30]. Additionally, the SASP can induce senescence cell non-autonomously in neighbouring cells (i.e. paracrine-induced senescence or bystander effect) or mediate cancer cell clearance by the immune system [31]. For this, cellular senescence has been widely regarded as an innately protective mechanism that restricts cancer cell proliferation and tumour growth [32, 33]. However, the paradigm of senescence as a tumour-suppressing mechanism has been challenged by studies showing that senescent cells and the SASP can represent a double-edged sword with serious negative effects in cancer and other diseases. In particular, there is mounting evidence showing that paracrine SASP signals can stimulate several pro-tumourigenic cellular and molecular processes such as cancer cell proliferation, progression to malignancy, immune system evasion, resistance to therapy-induced apoptosis, angiogenesis, formation and maintenance of metastatic niches, as well as increased cell invasiveness, migration and epithelial-to-mesenchymal transitions (EMT) [34–41], and even induce tumour formation cell non-autonomously [42, 43]. We refer the reader elsewhere for comprehensive reviews on the pro-tumourigenic activities of senescence and the SASP [30, 44–46].

The cell non-autonomous origin of some tumours stands in stark contrast to traditional models of carcinogenesis [47–49]. A review of the available evidence supporting this scarcely discussed mechanism could provide further insights into the role of senescence in cancer. In this manuscript, we discuss studies on senescence and the SASP which have improved our understanding of the origins and biology of a paediatric pituitary tumour known as adamantinomatous craniopharyngioma (ACP). We describe two genetically engineered mouse models of ACP and present evidence supporting a cell non-autonomous model of tumour formation driven by senescence. We further explore the literature to discuss existing examples of the widely unexplored phenomenon of paracrine tumour initiation and highlight studies that have also shown a major role for senescence and the SASP in this process.

## Adamantinomatous craniopharyngioma and mouse ACP models

### Human adamantinomatous craniopharyngioma (ACP): clinical aspects and pathology

Craniopharyngiomas (CPs) are benign epithelial tumours (WHO grade 1) of the sellar region, which is an anatomical structure located between the hypothalamus and the cranial base. CPs represent between 1.2 and 4.6% of all intracranial tumours, with an incidence of 0.5–2.5 new cases per 1 million population per year [50, 51]. There are two subtypes of CPs, the papillary and the adamantinomatous (PCP and ACP, respectively), which differ in their clinical, histological and molecular features [52]. Because of the proven relevance of senescence in ACP, in this review, we will focus only on this tumour type.

ACPs represent the most common non-neuroepithelial intracranial tumours in children and young adults [50, 53]. They are difficult to manage and can behave aggressively in the clinic. Additionally, treatments are non-specific (i.e. maximal safe surgical resection avoiding damage of the hypothalamus and visual pathways, followed by radiotherapy), non-curative and associated with high morbidity [53–57]. This morbidity is due to the tumour’s tendency to invade surrounding structures such as the pituitary, hypothalamus and optic chiasm. Consequences of both tumour growth and its treatment include pan-hypopituitarism with multiple neuroendocrine deficiencies, blindness and hypothalamic damage, which usually leads to obesity, subsequent type-2 diabetes and cardiovascular disease [58–60]. Furthermore, reduced psychosocial and neurocognitive function are common in survivors, mostly in patients of younger age [58, 61]. All of these comorbidities lead to poor quality of life and increased long-term mortality in survivors [62].

**Fig. 1:**
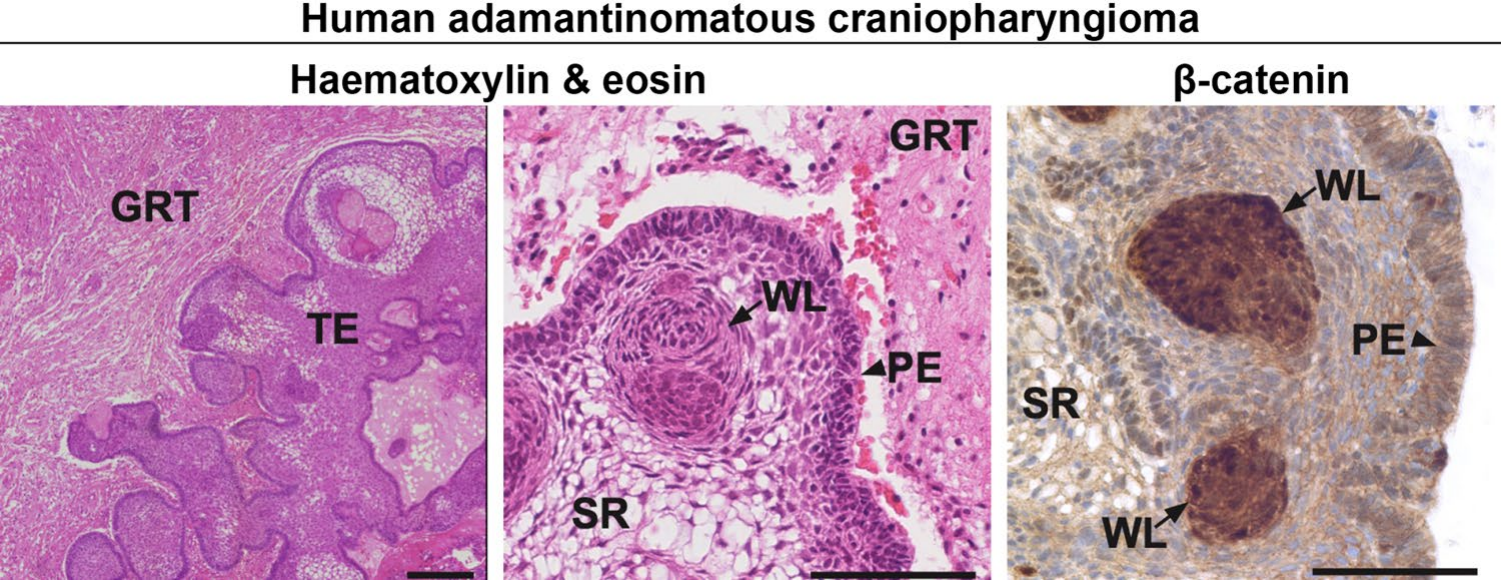
Histopathology of human adamantinomatous craniopharyngioma (ACP). *TE* tumour epithelium, *GRT* glial reactive tissue, *PE* palisading epithelium, *SR* stellate reticulum, *WL* whorl-like epithelial cell groups. Immunostaining for β-catenin showing nucleo-cytoplasmic accumulation in cells of the WL, whilst the rest of the tumour cells show normal membranous staining. Scale bar 200 μm. The figure is adapted from Martinez-Barbera JP, Andoniadou CL (2020) Biological Behaviour of Craniopharyngiomas. Neuroendocrinology 1–8, with permission of S. Karger AG, Basel

The histomorphological features of ACPs are well defined (Fig. 1). The tumour epithelium is surrounded by glial reactive tissue that is comprised of non-tumoural cells such as astrocytes, immune cells and fibroblasts [63]. The tumours themselves are usually comprised of solid and cystic components [64]. The solid part includes epithelial tumour cells, organised in well-defined structures such as the palisading epithelium, the stellate reticulum and whorl-like structures. The epithelial component of these tumours shows no sign of neuroendocrine differentiation (i.e. lack of expression of pituitary hormones, cell-lineage markers or neuroendocrine markers like synaptophysin). Additional solid components include wet keratin (i.e. eosinophilic areas of keratinised cells without nuclei) and calcification foci. In addition to these solid structures, ACP tumours usually contain one or multiple cysts filled with a dark fluid enriched in inflammatory mediators and lipids [65–67].

**Fig. 2:**
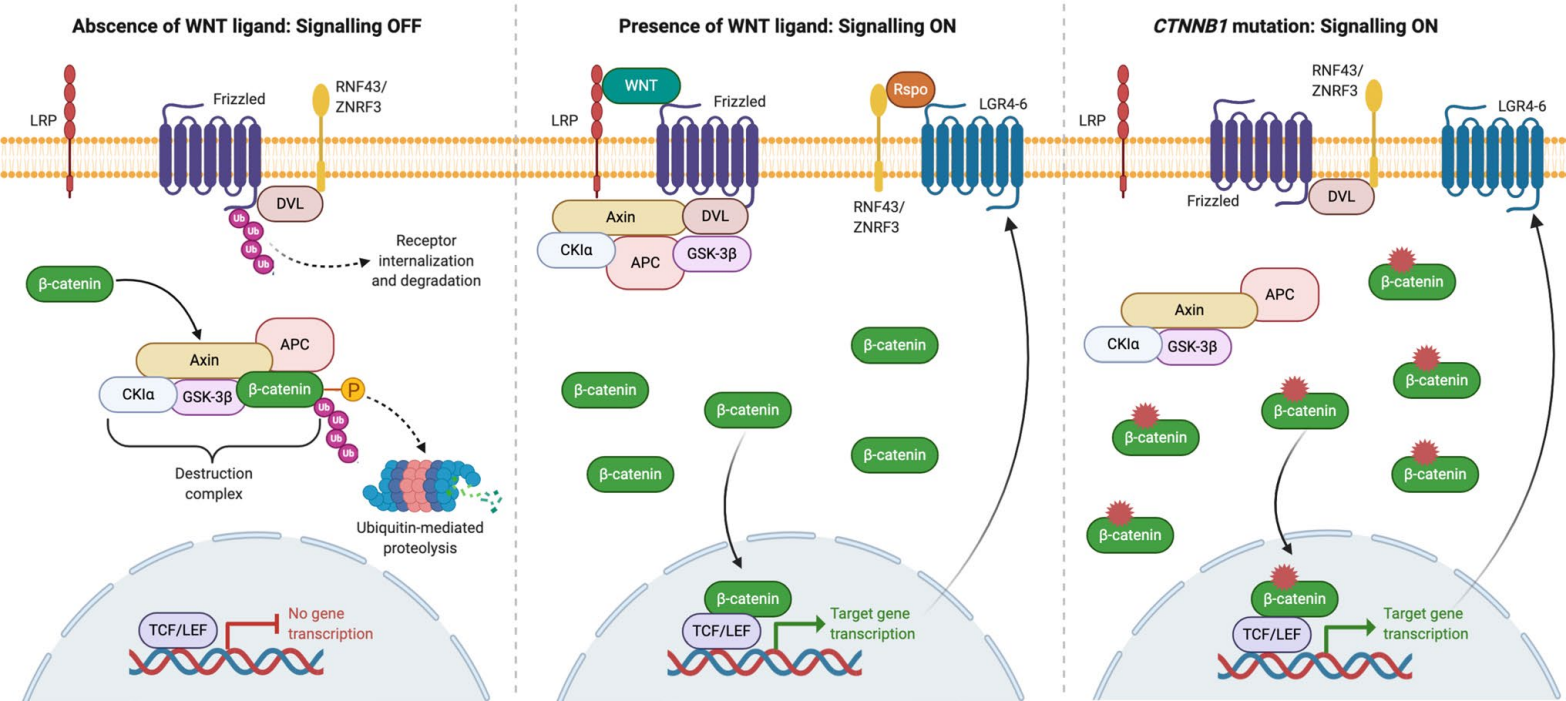
Schematic of main components of the canonical WNT signalling pathway. In the absence of WNT ligands, β-catenin, (encoded by the *CTNNB1* gene) is normally recruited in a destruction complex containing several proteins including APC (adenomatous polyposis coli), AXIN, CKIα (casein kinase 1 alpha) and GSK3β (glycogen synthase kinase 3β). This results in β-catenin phosphorylation of specific amino acids encoded by *CTNNB1* exon 3 and protein degradation by the ubiquitin–proteasome pathway. Consequently, levels of β-catenin protein concentration are low in the cytoplasm and nucleus, hence keeping the target genes in a repressed state. At the same time, two surface E3 ubiquitin ligases, RNF43 and ZNFR3, regulate levels of the WNT ligand–receptor Frizzled through its ubiquitination which leads to its endosomal internalization and degradation. Binding of WNT ligands to their receptor, Frizzled, leads to the formation of a complex alongside coreceptors LRP and Dishevelled (DVL), which captures and disassembles the β-catenin destruction complex and thus prevents β-catenin phosphorylation and degradation. This causes protein stabilization, nucleo-cytoplasmic accumulation of β-catenin and the activation of target genes. Examples of WNT target genes encode for LGR receptors (LGR4-6), which upon binding of R-spondins (Rspo), recruit the RNF43/ZNFR3 complex and, therefore, allows the accumulation of Frizzled in the membrane. This leads to positive feedback and amplification of the WNT signalling pathway. Mutations in exon 3 of *CTNNB1*, containing the regulatory amino acids of β-catenin responsible for its degradation, prevent β-catenin phosphorylation by the destruction complex. This leads to its nucleo-cytoplasmic accumulation and constitutive overactivation of the WNT/β-catenin pathway even in the absence of WNT ligands and R-spondins. Created with BioRender.com

**Fig. 3:**
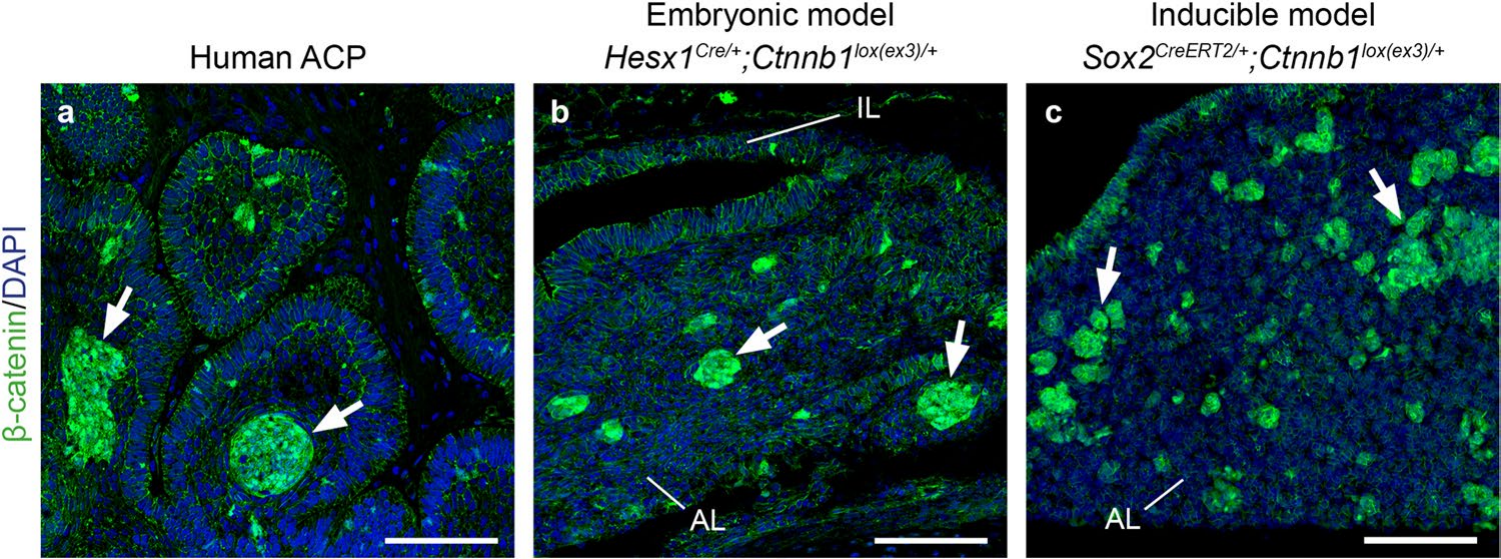
Human adamantinomatous craniopharyngioma (ACP) and ACP murine models contain nucleo-cytoplasmic β-catenin-accumulating cell clusters. **a** Immunofluorescent staining in human ACP showing the nucleo-cytoplasmic accumulation of β-catenin in cell groups known as “clusters” (arrows), a defining characteristic of these tumours. **b** Expression of oncogenic β-catenin in Rathke’s Pouch progenitors leads to the formation of clusters in a *Hesx1* ^*Cre/*+^; *Ctnnb1* ^*lox(ex3)/*+^ pre-tumoural pituitary. **c** Clusters also form upon inducible expression of oncogenic β-catenin in adult pituitary stem cells in *Sox2* ^*CreERT2/*+^; *Ctnnb1* ^*lox(ex3)/*+^ mice. A 16-week post-tamoxifen induction pituitary is shown. Scale bars 100 μm. *AL* anterior lobe, *IL* intermediate lobe. The figure is reproduced with permission from Carreno G, Gonzalez-Meljem JM, Haston S, Martinez-Barbera JP (2016) Stem cells and their role in pituitary tumorigenesis. Mol Cell Endocrinol 445:27–34

Molecularly, ACPs are driven by the overactivation of the WNT/β-catenin signalling pathway [68, 69]. This pathway is heavily involved in normal development and physiology as well as in cancer [70]. Figure 2 depicts a schematic and description of the pathway. Research from the last 2 decades has demonstrated that mutations in exon 3 of *CTNNB1*, the gene encoding for β-catenin, are the most common molecular alterations associated with ACP tumourigenesis [68, 69]. These mutations are predicted to prevent protein degradation and cause nucleo-cytoplasmic accumulation of β-catenin and activation of the pathway [71]. In agreement, immunohistochemistry against β-catenin has shown the presence of sporadic epithelial tumours cells with cytoplasmic and nuclear staining, either dispersed throughout the tumour or grouped in whorl-like epithelial structures (also known as clusters) (Figs. 1, 3) [72]. Despite β-catenin accumulation being restricted to a minority of cells, *CTNNB1* mutations have been identified in all of the epithelial tumour cells in a large cohort of ACPs by combining laser capture microdissection with deep sequencing [73]. Three-dimensional imaging of human ACP tumours has revealed that these β-catenin-accumulating cell clusters are located within finger-like protrusions of tumour epithelium that invade the brain and surrounding structures, suggesting a potential role in tumour invasion [74]. Importantly, murine studies have demonstrated that mutations in *CTNNB1* are tumour drivers and provided important insights into the role of the nucleocytoplasmic β-catenin cell clusters in ACP tumourigenesis (see below) [43, 75]. The cellular origin of human ACP is still a matter of debate, with the most prominent hypothesis being that it arises from embryonic oral ectoderm and in particular from remnants of Rathke’s pouch epithelium, a proposition derived from the observation of a common expression of certain cytokeratins between ACPs and oral epithelium [71, 76, 77]. In support of this, a recent RNA sequencing study found that human ACPs share a common transcriptional profile with tissues present during normal tooth development [67].

### Mouse models of ACP: insights into tumour initiation and pathogenesis

**Fig. 4:**
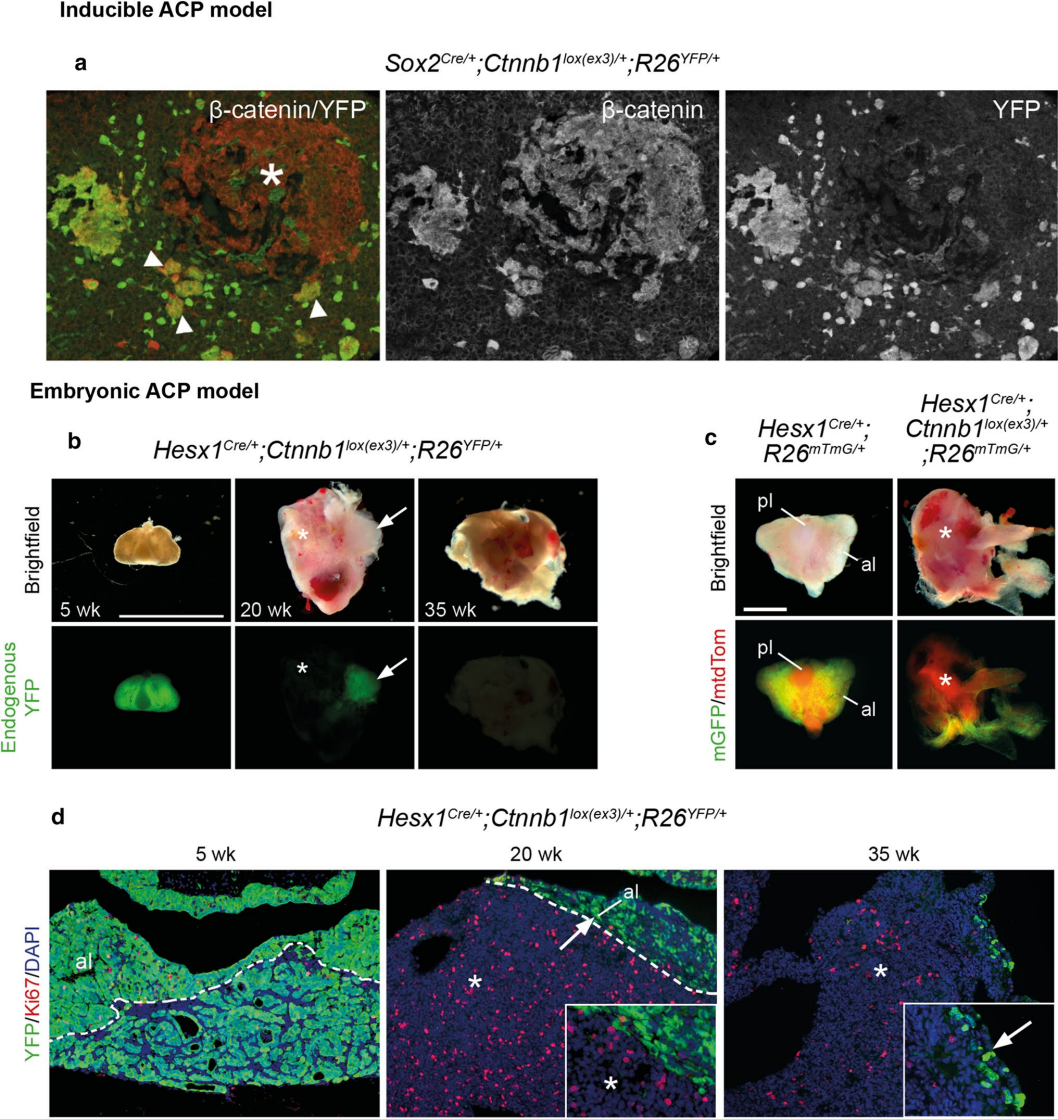
Lineage tracing in mouse ACP models shows the cell non-autonomous origin of tumours. Mouse ACP models were crossed with *R26* ^*YFP/*+^ lineage reporter mice which allows labelling of cells and their descendants upon expression of Cre recombinase. **a** Double immunofluorescent staining in *Sox2* ^*CreERT2/*+^; *Ctnnb1* ^*lox(ex3)/*+^; *R26* ^*YFP/*+^ pituitaries showing cell clusters (arrowheads) and a large tumoural lesion (asterisk) that accumulate β-catenin. The tumour cells (asterisk) do not express the lineage reporter YFP, indicating they are not descendants of SOX2 + stem cells. Note that the clusters (arrowheads) co-express nucleocytoplasmic β-catenin and YFP, demonstrating that they derive from SOX2 + stem cells. **b** In *Hesx1* ^*Cre/*+^; *Ctnnb1* ^*lox(ex3)/*+^; *R26* ^*YFP/*+^ mice, most cells of the anterior lobe of the pituitary descend from HESX1 + Rathke’s pouch precursor cells, as shown by YFP expression in a 5-week-old pituitary. After a period of latency, pituitary tissue is displaced by developing tumour tissue that does not express YFP. Scale bar 5 mm. **c** The absence of Cre-mediated recombination in the tumours is further demonstrated using the mT/mG dual reporter mouse line, in which unrecombined cells express membrane TdTomato protein (red) while pituitary-lineage cells express GFP (green). Note that in the *Hesx1* ^*Cre/*+^; *R26* ^*mTmG/*+^ control pituitary, the anterior lobe (al) tissue is green (recombined) and the posterior lobe (pl) is red (unrecombined since the pl is not derived from the Hesx1 lineage). In an ACP *Hesx1* ^*Cre/*+^; *Ctnnb1* ^*lox(ex3)/*+^; *R26* ^*mTmG/*+^ mouse tumour, most of the tumour cells express TdTomato. Scale bar 1 mm. **d** Double immunofluorescent staining against the proliferation marker Ki67 and YFP revealing that although most of the cells in the pituitary anterior lobe (al) of a 5-week-old ACP embryonic model are YFP + ve, the tumours in a 20-week-old mouse develop from YFP-ve cells that show a high proliferative activity (asterisk). In very advanced tumours (35 weeks) most of the YFP + ve cells are missing and only sporadic cells are detected in the periphery. Panel a is adapted with permission from Andoniadou CL, Matsushima D, Mousavy-gharavy SN, et al. (2013) The Sox2 + population of the adult murine pituitary includes progenitor/stem cells with tumour-inducing potential. Cell Stem Cell 13:433–445. **b**–**d** are adapted from Gonzalez-Meljem JM, Haston S, Carreno G, et al. (2017) Stem cell senescence drives age-attenuated induction of pituitary tumours in mouse models of paediatric craniopharyngioma. Nat Commun 8:1819, which is an open-access article licensed under a Creative Commons Attribution 4.0 International License

Two genetically engineered mouse models of ACP have been developed by expressing oncogenic β-catenin in either HESX1 + embryonic precursors of the developing pituitary (embryonic model; *Hesx1* ^*Cre/*+^; *Ctnnb1* ^*lox(ex3)/*+^ mouse line) or in SOX2 + adult pituitary stem cells (inducible model; *Sox2* ^*CreERT2/*+^; *Ctnnb1* ^*lox(ex3)/*+^ mouse line) (Fig. 4a, b, respectively) [43, 75]. These mouse models utilise the Cre/loxP technology to induce the expression of a murine oncogenic form of β-catenin that is functionally equivalent to those identified in human ACPs. Specifically, Cre recombinase expression in either *Hesx1* or *Sox2*-expressing cells leads to the deletion of exon 3 from the *Ctnnb1lox(Ex3)* allele, which then codes for a mutant version of β-catenin missing several important regulatory amino acids located in the N-terminal end of the protein. Degradation of this mutant β-catenin is impaired, as the protein cannot be phosphorylated by its destruction complex but can still activate transcription of target genes (Fig. 2). In agreement, both of these ACP murine models show over-activation of the WNT/β-catenin pathway (e.g. expression of target genes such as *Axin2* and *Lef1*) in the developing (embryonic) or adult (inducible) pituitary leading to the formation of tumours resembling human ACP [72, 78].

In the embryonic model, the expression of oncogenic β-catenin is driven into early precursors of Rathke’s pouch (RP) expressing the homeobox transcription factor *Hesx1* [75]. RP is the primordium of the anterior pituitary and these precursors give rise to all of the pituitary hormone-producing cells (e.g. somatotrophs expressing growth hormone and corticotrophs expressing adrenocorticotrophic hormone, among others) [79]. Sporadic cells and cell clusters accumulating nucleo-cytoplasmic β-catenin are observed in the developing pituitary soon after the initial expression of oncogenic β-catenin in RP precursors and prior to any sign of cell transformation or tumour development (Fig. 3). Mice are born without pituitary tumours but the clusters are detectable for the first several weeks of postnatal life [80]. Analysis of tumour growth dynamics in the embryonic model has revealed a latency period of approximately 18 weeks from birth until the appearance of proliferative tumours [81]. It is possible that human ACPs follow similar a behaviour as there is a bimodal peak distribution for the age at diagnosis; first at 5–15 years (paediatric ACP) and at 45–60 (adult ACP), while a number of neonatal and embryonic cases have also been reported [58, 82–85]. As in human ACP, cells accumulating β-catenin are a minority despite the DNA mutation (i.e. *Ctnnb1* exon 3 deletion) being present throughout the developing pituitary cells as shown by laser capture microdissection and PCR [75]. Mouse tumours contain solid and cystic components, do not express markers of neuroendocrine differentiation and show histological and imaging features resembling human ACP [81].

An interesting finding from the analysis of the embryonic model is that if oncogenic β-catenin is expressed in committed or differentiated pituitary embryonic cell types, rather than in *Hesx1*-expressing undifferentiated precursors, clusters do not form and tumours never develop [75]. Because the RP precursors are multipotent, this finding suggests that tumour formation requires a stem-like cell to express oncogenic β-catenin. This possibility has been further explored in the inducible model. Expression of oncogenic β-catenin in SOX2 + ve cells of the postnatal pituitary, which contain *bona fide* organ-specific stem cells results in the initial formation of β-catenin-accumulating cell clusters and subsequent development of ACP-like tumours after a latency period of 3–6 months (Fig. 4a) [43]. In conclusion, data from these mouse models support the idea that the *CTNNB1* mutations identified in human ACP are drivers of tumourigenesis and have provided evidence that ACP likely originates from embryonic RP precursors. Additionally, the dynamics of tumour development suggests that the formation of β-catenin-accumulating cell clusters precede cell transformation and tumour initiation.

### Paracrine tumourigenesis in the ACP mouse models

An interesting question that has been investigated using the ACP murine models is to interrogate the fate of the cells expressing and accumulating oncogenic β-catenin. This can be addressed in mice by permanently labelling these cells and their progeny with the expression of a fluorescent reporter protein (e.g. yellow fluorescent protein, YFP), an approach known as genetic lineage tracing [86]. Genetic tracing has been initially carried out in the inducible model to reveal that the ACP-like tumours do not derive from SOX2 + pituitary stem cells expressing oncogenic β-catenin [43]. Instead, targeted SOX2 + stem cells give rise to β-catenin-accumulating cell clusters, while the tumours originate from a different cell lineage as they do not express the lineage reporter, nor do they contain a *Ctnnb1* exon 3 deletion (Fig. 4a). These findings are rather unexpected, especially when considering that in other systems (e.g. intestinal tumours), stem cells have been shown to become the tumour cell-of-origin when targeted with similar oncogenic mutations that result in the overactivation of the WNT/β-catenin signalling pathway [87, 88]. In agreement, it has been shown that SOX2 + pituitary stem cells can form tumours cell autonomously when YAP/TAZ signalling is enhanced [89]. The cell non-autonomous origin of the ACP-like tumours has been further corroborated in the embryonic model by lineage tracing (Fig. 4b–d) and DNA sequencing, which have shown that the tumours derive from a different cell lineage and contain novel somatic mutations not present in the germline [42]. Therefore, the expression oncogenic β-catenin in either SOX2 + adult stem cells or HESX1 + embryonic progenitors leads first to the formation of clusters, which are similar to those found in human ACP, and then promote tumour formation in a cell non-autonomous manner. Importantly, in both ACP models and in human ACP, these clusters are non-proliferative and express a multitude of growth factors (e.g. WNTs, FGFs, BMPs, EGF, among others) and inflammatory mediators (e.g. IL1, IL6, CXCL1, CXCL20, among others) [42, 43, 90]. Of note, recent research indicates that SOX2 + stem cells in the normal pituitary have an important paracrine signalling function by mediating the expansion of neighbouring committed progenitor cells, suggesting that oncogenic β-catenin and the senescence programme (discussed below) may consolidate the secretory phenotype of SOX2 + stem cells [91]. These data support a model of paracrine tumourigenesis, whereby non-dividing cluster cells are bestowed with the capacity to initiate tumours in a cell non-autonomous manner.

## The role of senescence and its paracrine signals in ACP

### Cellular senescence mediates paracrine tumourigenesis in mouse ACP models

Molecular profiling and genetic approaches have shown that these β-catenin accumulating clusters are enriched in senescent cells in both mouse and human ACP. Specifically, human and mouse cluster cells exhibit several hallmark features of senescence: (1) they are viable and non-proliferative (Ki67 and EdU negative); (2) express cell cycle inhibitors (e.g. p21^CIP1^); (3) exhibit DNA damage and activation of a DNA damage response; (4) have an enlarged lysosomal compartment (e.g. they show elevated expression of GLB1, the enzyme responsible for the widely employed senescence-associated β-galactosidase staining); (5) activate the NF-κB pathway and a SASP [42]. Corroborating the expression data above, unbiased molecular analyses have revealed that the cluster cells are analogous cellular structures in human ACP and both mouse ACP models, which share a common signature of senescence and SASP activation [42].

**Fig. 5:**
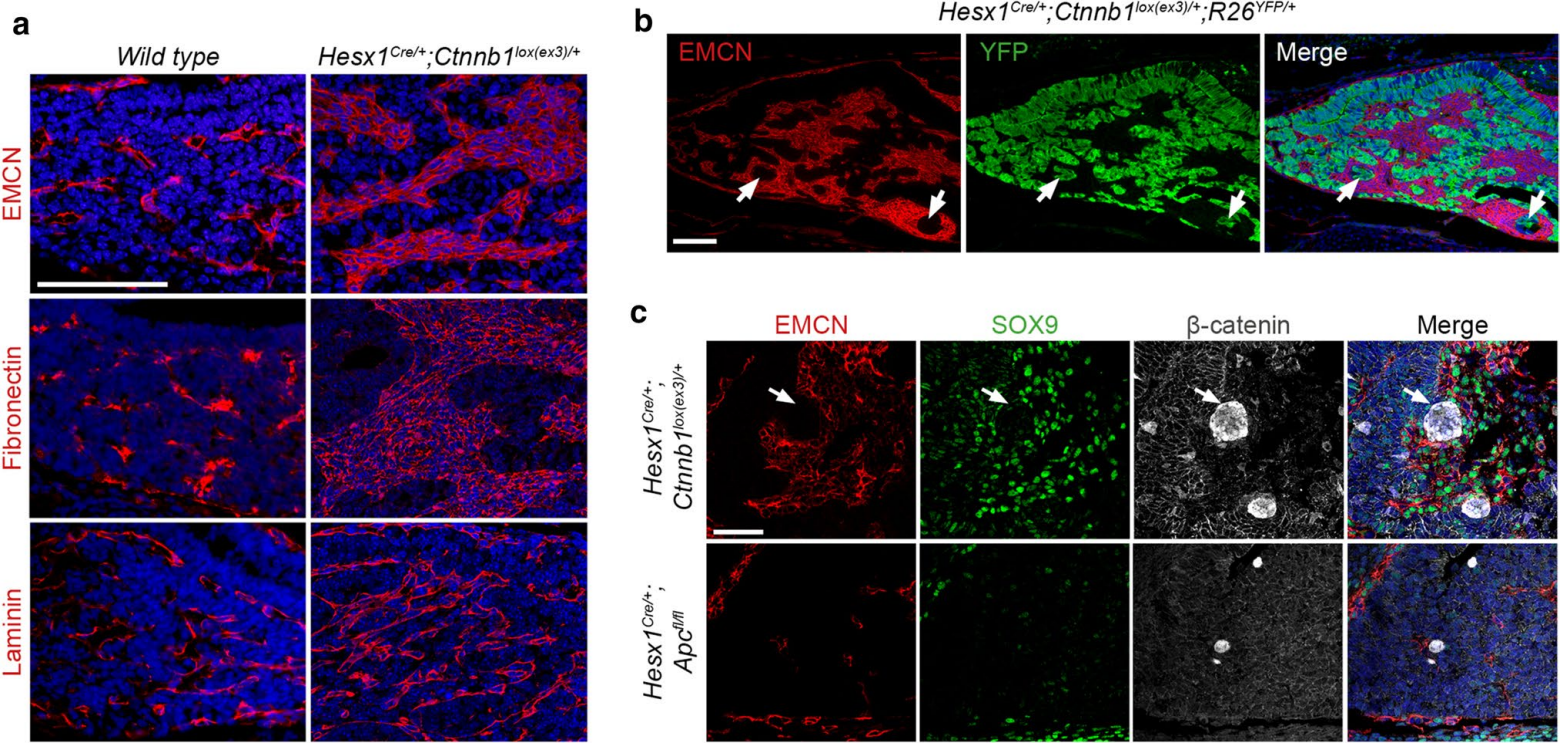
The β-catenin accumulating senescent cell clusters modify the tumour microenvironment (TME) in the pre-tumoural pituitary of the ACP embryonic model. **a** Immunostaining against fibronectin, laminin and endomucin (EMCN) showing TME alterations prior to tumour initiation in the ACP mouse model. Scale bar 100 μm. **b** Double immunostaining for the lineage tracing reporter YFP showing an expanded population of EMCN-expressing cells that is not derived from the Hesx1 cell lineage targeted with oncogenic β-catenin. Note that clusters of YFP + ve cells are often surrounded by EMCN + ve cells (arrows). Scale bar 100 μm. **c** Triple immunostaining showing that in the context of oncogenic β-catenin (*Hesx1* ^*Cre/*+^; *Ctnnb1* ^*lox(ex3)/*+^ pituitary, top panel), large numbers of EMCN + ve cells also co-express SOX9 and interact closely with the senescent clusters (arrows). However, in the context of wild type β-catenin (loss of tumour suppressor *Apc* in *Hesx1* ^*Cre/*+^; *Apc* ^*fl/fl*^ pituitaries), senescent clusters are smaller and show an attenuated SASP that fail to induce changes in the TME. Of note, *Hesx1* ^*Cre/*+^; *Apc* ^*fl/fl*^ do not develop tumours (see text). Scale bar 50 μm. The figure is adapted from Gonzalez-Meljem JM, Haston S, Carreno G, et al. (2017) Stem cell senescence drives age-attenuated induction of pituitary tumours in mouse models of paediatric craniopharyngioma. Nat Commun 8:1819, which is an open-access article licensed under a Creative Commons Attribution 4.0 International License

We have also reported that the presence of an activated SASP results in substantial microenvironmental changes in the pre-tumoural pituitary of the embryonic model. These changes include an excess production of ECM proteins (Fig. 5a), as well as the recruitment of YFP-ve (i.e. not targeted with oncogenic β-catenin) proliferative cells that coexpress the endothelial marker endomucin (EMCN) and the stem cell marker SOX9, which closely interact with the senescent clusters (Fig. 5b, c). Although these changes are less apparent in the inducible model, the presence of senescent β-catenin-accumulating cell clusters in this model also leads to increased proliferation in nearby non-cluster cells (i.e. increased Ki67 mitotic index) [42]. Notably, the close interaction of clusters with SOX9-expressing cells occurs in both mouse models as well as in human ACP [43, 75].

The function of senescent cluster cells in ACP tumourigenesis has been investigated in mice using two genetic approaches. First, in the inducible model, it has been shown that the expression of oncogenic β-catenin in SOX2 + pituitary stem cells at different ages results in a significant reduction in tumour formation when comparing induction in aged (6–9 months old) vs. young (4–6 weeks old) mice. This reduction in tumour burden is associated with a significant decrease in the senescence/SASP response in the clusters of the aged mice. Specifically, pituitaries from older mice contain smaller clusters and reduced expression of SASP factors, while reduced numbers of proliferating cells are also observed surrounding the clusters [42].

A second approach has taken advantage of the fact that deletions or inactivating mutations in the genes and proteins of the β-catenin destruction complex, such as *Apc* (adenomatous polyposis coli) (Fig. 2), also lead to the over-activation of the WNT/β-catenin pathway and tumour formation (e.g. in intestinal cancers in mouse and humans) [87, 88]. However, the deletion of *Apc* in both *Hesx1*-expressing embryonic precursors, or SOX2 + adult stem cells, completely fails to generate ACP-like tumours. Detailed histological and molecular analyses have revealed that deletion of *Apc* also leads to the formation of β-catenin clusters that activate downstream targets of the WNT pathway and express markers of senescence. However, these clusters are smaller in size in comparison to those carrying oncogenic β-catenin and, importantly, show a drastically reduced expression of SASP factors [42]. In addition to an absence of tumour induction, *Apc*-null pituitaries do not display the microenvironmental alterations that usually precede tumour growth, such as the aberrant expression of ECM markers or the recruitment of a population of proliferating non-oncogene targeted (YFP-negative) endothelial-like cells expressing SOX9 (Fig. 5c). Therefore, the attenuation of the paracrine activities of the senescent cluster cells in the aged inducible model and in *Apc*-deficient mice results in reduced tumour induction [42].

**Fig. 6:**
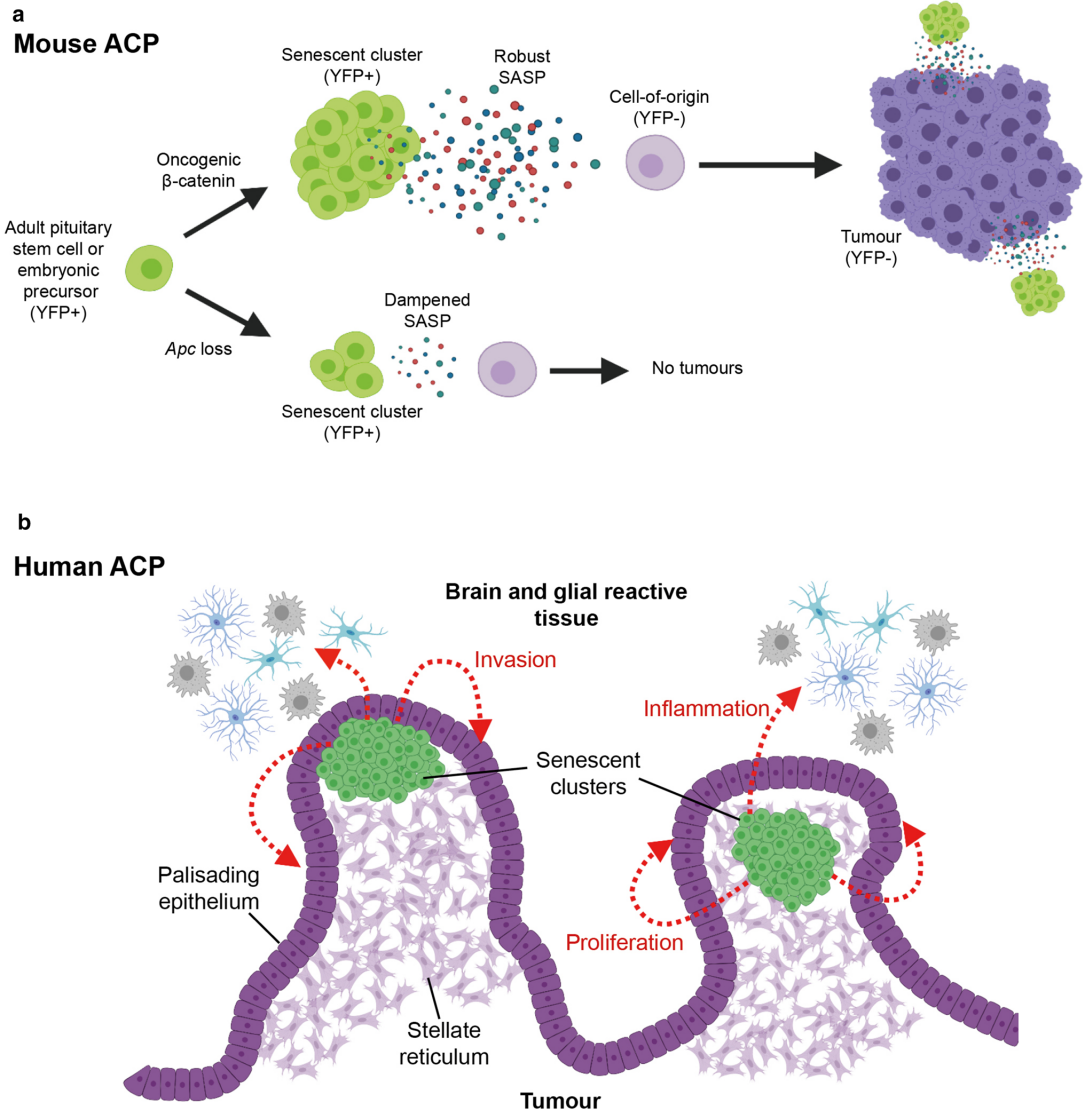
Models depicting the role of senescent cells in mouse and human ACP. **a** Oncogenic β-catenin expression in either in SOX2 + adult pituitary stem cells or Hesx1 pituitary embryonic progenitors leads to the formation of nucleocytoplasmic β-catenin-accumulating clusters and SASP activation with expression of several cytokines, chemokines and growth factors. Persistent and robust SASP promotes cell transformation of a non-targeted cell (i.e. not expressing oncogenic β-catenin or YFP) in a paracrine manner. In this model SASP-mediated activities of the clusters are required for either tumour initiation, progression or both. **b** Senescent clusters in human ACP (green cells) are usually found at the base of finger-like tumour projections that invade the brain. The factors secreted by the clusters are proposed to promote tumour cell proliferation of the palisaded epithelium and epithelial bending resulting in tumour invasion. Signals may also promote inflammation in the glial reactive tissue. Created with BioRender.com

In human ACP, the location of the senescent clusters within the finger-like tumour protrusions invading the brain suggests that SASP activities may promote epithelial cell proliferation and invasion [74]. This is further supported by the molecular similarities between human clusters and the enamel knot, a signalling hub controlling epithelial bending and proliferation during tooth formation [67]. In addition, the inhibition of the MAPK pathway in human ACP explant cultures results in reduced proliferation and increased apoptosis, while several ligands capable of activating the MAPK pathway are expressed in both human and murine senescent clusters (e.g. FGFs and EGF) [67, 90]. Together, evidence from mouse and human studies strongly suggests that senescence plays an essential role in tumour initiation in the murine models and in tumour growth, invasion and inflammation in human ACP (Fig. 6).

Paracrine tumourigenesis and senescence-mediated paracrine tumourigenesis mechanisms have not yet been described in other pituitary tumours. In the case of pituitary adenomas, cellular senescence has been shown to restrict tumour cell proliferation and is thought to underlie the almost invariable benign nature of pituitary tumours [92–97]. There are, however, interesting findings suggesting that paracrine SASP signalling might also contribute to pituitary adenoma pathogenesis. For example, it has been shown that senescent cells in somatotroph adenomas also secrete growth hormone (GH) as part of their SASP and that GH and growth hormone releasing hormone (GHRH) are inducers of DNA damage and genomic instability in normal pituitary cells [98–100]. Additionally, IL6 is a crucial SASP factor that is normally secreted by pituitary folliculostellate cells, and while it has been shown to induce cellular senescence in adenoma cells, it has also been shown to promote pituitary cell proliferation and to be required for tumour induction in a somatotroph adenoma transplant model [101, 102]. These findings suggest that senescence and the SASP could play a dichotomous role in pituitary adenomas by restricting cell proliferation and preventing the onset of malignancy in established tumours, while promoting the acquisition of genomic instability and the formation of tumour permissive microenvironments.

## Paracrine and senescence-induced tumorigenesis

### Evidence for paracrine tumorigenesis

Current and widely supported theories of carcinogenesis such as the cancer stem cell/hierarchical model, the genetic/stochastic evolution model and more recent unifying propositions imply that a tumour (or each subclonal population within a tumour) is formed by cells that are descendants of the cell originally targeted by an initiating oncogenic insult [47–49]. On the other hand, the observation that tumours can arise cell non-autonomously, as in mouse models of ACP, stands in stark contrast to traditional theories of the origin of cancer. There are, however, several experimental examples showing evidence that cell transformation and tumour initiation mechanisms do not necessarily follow traditional paradigms (Table 1).

**Table 1:** List of studies showing evidence of paracrine tumourigenesis

Organ/system	Implicated signal	Model and species	Signalling cell/tissue	Responding cell/tissue	Inducer^a^	Tracing^b^	Evidence of paracrine initiation/transformation	References
Blood/bone marrow	JAG1	Mouse in vivo model of MPD	Embryonic hepatocytes	Embryonic myeloid progenitors	Constitutive deletion of IκBα	No	Phenotype does not develop when mutation is targeted to responding cells. Coculture experiments with hepatocytes	[184]
	TNFA	Mouse in vivo model of MPD	Bone marrow	GMPs	Constitutive deletion of retinoic acid receptor	No	MPD induced upon transplantation of wild type BM cells into mutant microenvironment and not when mutant BM cells were transplanted into wild type microenvironment	[185]
	N/D	Mouse in vivo model of MPD	Bone marrow stromal cells	Myeloid progenitors	*Mib* deletion in the bone marrow stroma	Yes	BM replacement experiments showed that mutation was required in the stromal compartment to induce wild type BM cells to form myeloid neoplasias	[186]
	N/D	Mouse in vivo model of MDS, AML and myeloid sarcoma	Osteolineage progenitors	Myeloid progenitors	Deletion of *Dicer1* in osteoprogenitors	Yes	BM replacement and coculture experiments show osteoprogenitors can induce myeloid neoplasia paracrinally. Intact *Dicer1* allele in non-targeted cells and presence of other genomic aberrations in neoplastic cells	[117]
	JAG1	Mouse in vivo AML model	Osteoblasts	Long-term repopulating hematopoietic stem cells	Activation of oncogenic β-catenin in osteoblasts	No	Presence of chromosomal aberrations and non-silent somatic mutations in myeloid cells. Autonomous cell growth after transplantation in irradiated BM	[118]
Brain	PDGFB	Mouse in vivo glioma model	Nestin + transfected cells	Non-targeted, recruited Olig2 + cells	Gene expression by viral transfection	Yes	Recruited cells can form tumours upon transplantation which could be serially passaged and become independent of PDGF signalling	[121]
	PDGFB	Human to mouse xenotransplant glioma model	Human glioma-stem like cells (SU3)	Mouse oligodendrocyte progenitor cells	Transplantation or coculture with human glioblastoma stem cells	Yes	Recruited cells form tumours upon transplantation in nude mice. Cells had increased proliferative and invasive capacity. Using donor cells with reduced PDGFB expression failed to induce tumours	[122]
	N/D	Mouse in vivo CNS hemangioblastoma model	Brain interstitial cells	Endothelial cells	Conditional activation of KRasG12V under the Rag1 promoter	Yes	Tumours formed by cells not carrying active version of the oncogene as determined by LCM and PCR	[187]
	N/D	Human to mouse xenotransplant glioma model	Human glioma-stem like cells SU3	Mouse BMDMSCs	Transplantation or coculture with human glioblastoma stem cells	Yes	Host BMDMSCs became highly proliferative and able to form transplantable tumours	[188]
	PDGFAA	Mouse in vitro glioblastoma model	N/A	Subventricular zone cells	Culture in the presence of PDGFAA	No	P53-null cells could eventually grow independently of PDGFAA, displayed chromosomal instability and formed tumours when transplanted into nude mice	﻿[189]
Breast	WNT1	Rodent in vitro coculture	Rat fibroblast cell line (Rat2)	Mouse mammary epithelial cell line (C57MG)	*Wnt1* gene expression by viral transfection	No	Loss of contact inhibition at confluence, increased cell density, alteration of cell morphology	[104]
	WNT1	Mouse in vitro coculture	Mouse embryonic fibroblast cell line (NIH 3T3)	C57MG cells	*Wnt1* gene expression by viral transfection	No	Alteration of cell morphology and increased cell density	[105]
	WNT11	Rodent in vitro coculture	Rat fibroblast cell line (Rat2)	C57MG cells	*Wnt11* gene expression by viral transfection	No	Loss of contact inhibition at confluence, increased cell density, alteration of cell morphology	[106]
	WNT1, WNT2, WNT3, WNT3A	Rodent in vitro coculture	Rat fibroblast cell line (RatB1a)	C57MG cells	Wnt ligand gene expression by viral transfection	No	Loss of contact inhibition at confluence, increased cell density, alteration of cell morphology	[107]
	HGF, TGFB1	Human to mouse xenotransplant mammary gland cancer model	Irradiated human mammary stromal cells cell line (RMF/EG)	Organoids derived from human mammary epithelial cells	*HGF* of *TGFB1* gene expression by viral transfection	No	Development of neoplastic histological features including hyperplasia, in situ ductal cancer and invasive carcinomas	[119]
	N/D	Rat in vivo mammary tissue recombination model	Mammary stroma (cleared fat pad)	Mammary epithelial cells	NMU treatment	No	Development of neoplasia and papillary carcinomas only when stroma was treated with carcinogen	[120]
Gastrointestinal	N/D	Mouse in vivo aggregation chimera intestinal tumorigenesis model	*Apc* ^*Min/*+^ mutant intestinal epithelium	Wild type intestinal epithelium	Constitutive mutant *Apc* allele	Yes	Lineage tracing in chimeras showed the formation of heterotypic tumours	[123]
	TNFA	Mouse in vivo intestinal-type gastric tumourigenesis model	Macrophages	Gastric epithelium	Constitutive overexpression of *Wnt1*, *Cox2* and prostaglandin E synthetase 1	Yes	Gastric tumour initiation depended on macrophage TNFA signalling which leads to the expansion of SOX2 epithelial progenitor cells	[124, 190]
Liver	FGF19	Mouse in vivo hepatocellular carcinoma model	Skeletal muscle	Pericentral hepatocytes	Transgenic expression of FGF19 in skeletal muscle	No	Formation of hepatocellular neoplasias. Increased cell proliferation, nuclear translocation of β-catenin and development of mutations leading to amino acid substitutions in the regulatory domain of the β-catenin gene	[128]
	FGF19	Mouse in vivo hepatocellular carcinoma model	N/A	Hepatocytes	Exogenous FGF19 delivered by injection or expressed by viral vector	No	In vivo formation of hepatocellular neoplasias. Absence of tumour formation with genetic ablation or antibody blockade of the FGF19 targets STAT3 and IL6	[129]
Prostate	HGF	Mouse in vivo* FSP1* ^*Cre*^; *Tgfbr2* ^*floxE2/floxE2*^ conditional knockout	Fibroblasts	Prostate and forestomach epithelial cells	Conditional knockout of *Tgfbr2* in fibroblasts	No	Formation of prostate intraepithelial neoplasias and invasive squamous cell carcinomas of the forestomach	[191]
	FGF10	Mouse in vivo prostate gland reconstitution model	Embryonic urogenital sinus mesenchyme	Prostate epithelial cells	FGF10 expression by viral transfection	Yes	Formation of multifocal prostatic intraepithelial neoplasia and prostatic adenocarcinoma some of which could be serially transplanted	[125, 126]
	FGF10	Mouse in vivo prostate gland reconstitution model	Embryonic urogenital sinus mesenchyme	Prostate epithelial cells	Overexpression of FGR2 in epithelial cells and FGF10 expression in mesenchyme by viral transfection	Yes	Formation of multifocal prostatic intraepithelial neoplasia and prostatic adenocarcinoma	[127]
Skin	FGF7 CXCL12	Mouse in vivo chemical carcinogen-induced skin cancer model	Targeted epidermis	Untargeted epidermis	Deletion of *Notch1* and DMBA/TPA treatment	Yes	Formation of papillomas formed by non-targeted cells as shown by lineage tracing and PCR	[133]
	N/D	Mouse in vitro coculture	Irradiated normal epidermis cell line (JB6)	Non-irradiated JB6 cells	Gamma radiation	Yes	Anchorage-independent growth in soft agar	[192]
	IL1A	Mouse in vivo transgenic epidermal papilloma model	Suprabasal transgene-positive cells	Basal transgene-negative cells and BMDCs	Expression of constitutively active MEK1 in suprabasal layer of epidermis under involucrin promoter aided by wounding	Yes	Spontaneous papilloma formation (9%) which is enhanced by wounding (40%). Cell proliferation is mostly from untargeted cells which was confirmed by lineage tracing in aggregation chimeras	[132]
	WNT3, WNT5A, WNT10B, WNT16	Mouse in vivo genetically engineered skin cancer model	K19 + hair follicle stem cells	Non-targeted hair follicle epithelial cells	Conditional activation of oncogenic β-catenin in K19 + hair follicle stem cells	Yes	Generation of hair follicle outgrowths and benign tumours composed of mutant and wild-type cells. Regrowth of hair follicles after laser ablation of dermal papilla cells	[135, 137]
	VEGF, IL6, CXCL1	Mouse in vitro and in vivo allograft Kaposi´s Sarcoma models	Endothelial cells expressing KSHV-vGPCR	Endothelial cells carrying KSHV latent genes	Expression of the GPCR encoded by KSHV which acts through PI3K–AKT–mTOR pathway	No	Grafted endothelial cells carrying latent KSHV genes only form tumours after being exposed to CM from cells expressing vGPCR	[193]
	N/D	Mouse in vivo skin tumorigenesis model	Hair follicle LGR5 + stem cells	Non-targeted epidermal cells	Activation of oncogenic β-catenin in Lgr5 + epidermal cells	Yes	Formation of neoplastic lesions resembling human pilomatrixoma	[136]
	N/D	Mouse in vivo chemical carcinogen-induced skin cancer model	Mutated K5 + keratinocytes	Non-targeted K5 + keratinocytes	DMBA/TPA treatment	Yes	Acquisition of Notch mutations that differed from the signature HRas mutation induced by DMBA	[134]
	N/D	Mouse in vivo model of keratoacanthoma and/or Bowen’s disease	Skin mesenchymal cells deficient in *CSL/RBP-Jk*	Non-targeted keratinocytes	Deletion of *CSL/RBP-Jκ* in skin mesenchyme	Yes	Tumour cells carried an intact RBP-Jκ allele but had other chromosomal alterations. Transplantation of normal keratinocytes alongside RBP-Jκ-deficient fibroblasts significantly enhanced tumour formation	[194]

*AML* acute myeloid leukaemia, *BM* bone marrow, *BMDCs* bone marrow-derived cells, *BMDMSCs* bone marrow-derived mesenchymal stem cells, *DMBA* dimethylbenz(a)anthracene, *FISH* fluorescent in-situ hybridization, *GMPs* granulocyte/macrophage progenitors, *KSHV* Kaposi’s sarcoma-associated herpes virus, *MDS* myelodysplastic syndrome, *MPD* myeloproliferative disorder, *N/A* not applicable, *N/D* not determined, *NMU* *N*-nitrosomethylurea, *LCM* laser-capture microdissection, *PCR* polymerase chain reaction, *TPA* 2-*O*-tetradecanoylphorbol-13-acetate, *vGPCR* viral G protein-coupled receptor

^a^“Inducer” column refers to conditions or stimuli that initiate cell transformation or tumour growth

^b^“Tracing” column refers to the use of any of the following techniques: gene expression reporters, lineage tracing reporters, immunophenotyping between host and donors carrying different CD45 alleles (CD45.1 and CD45.2), sex chromosome detection by FISH in cells derived from host and donors of different sexes and PCR genotyping of tumour/normal tissue regions either by scraping or LCM

Around 3 decades ago, in vitro experiments had already shown that paracrine or autocrine exposure of naïve cells to secreted developmental growth factors of the WNT and FGF families could initiate pre-malignant phenotypical changes, including: (1) ability to grow without attachment in agar plates (a sign of cell transformation); (2) alteration of cell morphologies; (3) increased proliferation and higher cell culture densities; (4) loss of contact inhibition of proliferation [103–109]. However, these initial studies did not address the capacity of the paracrinally “transformed cells” to generate tumours in vivo. Although research conducted over the last decades has established the importance of mutations and deregulation of these signalling pathways in several cancers [110–113], the exact relationships between a tumour’s cell-of-origin (also known as tumour-initiating cell), oncogenic driver mutations and the source of paracrine protumourigenic signals have not been widely explored until recently.

In vivo, combinations of different methods such as lineage tracing, cell ablation, DNA sequencing, laser-capture microdissection, chimera aggregations and cell transplantation have been instrumental in providing evidence of paracrine tumourigenesis; while it is important to note that these have also been used to support the validity of cell autonomous mechanisms in some in vivo cancer models [114–116]. For example, two notable studies used bone marrow replacement experiments in genetically engineered mouse models to show that the loss of *Dicer1* or the activation of oncogenic β-catenin, specifically in osteoprogenitors or osteoblasts, leads to the development of myelodysplastic syndromes and acute myeloid leukaemias in a paracrine manner. Interestingly, they show that the tumour cell of origin does not carry the targeted mutations (i.e. *Dicer1* or β-catenin) and instead display novel mutations and genomic aberrations [117, 118].

In breast cancer studies, most experiments so far have used in vitro cocultures to show the capacity of paracrine WNT signalling to transform mammary epithelial cell lines [104–107]. However, a study has shown that xenotransplanted mammary epithelial cell organoids required HGF and TGB1 secreted by irradiated stromal cells in order to form tumours in nude mice [119]. Likewise, another study has shown the requirement of mammary stroma exposed to the chemical carcinogen *N*-nitrosomethylurea (NMU) for the induction of mammary epithelial tumours [120].

In regard to brain cancer, lineage tracing and transplantation experiments have demonstrated an important role for paracrine PDGFB signalling in the recruitment of untargeted oligodendrocyte host cells to induce the formation of gliomas [121, 122]. Similarly, a combination of lineage tracing with chimera aggregations has shown the recruitment of untargeted cells during the formation of polyclonal intestinal tumours [123]. Likewise, combining lineage tracing and bone marrow transplantations has revealed the requirement of macrophage-secreted TNF for the initiation of gastric tumours [124] and that of FGF10 paracrine signalling between the urogenital mesenchyme and prostatic epithelia for the formation of prostatic neoplasias [125–127]. FGF signalling has also been implicated in cell non-autonomous tumourigenesis in the liver, as virally-delivered FGF19 or skeletal muscle-derived FGF19 were shown to be required for the induction of hepatocellular carcinomas in mice [128, 129].

In skin models of cancer, it is known that certain oncogenic driver mutations require wounding-derived inflammatory signals to initiate tumourigenesis [130, 131]. Interestingly, lineage tracing in a transgenic skin papilloma model has shown that activating constitutive MAPK signalling in suprabasal epidermal cells, in addition to skin wounding, can induce IL1A-driven hyperproliferation of non-targeted epidermal basal cells, which then formed the bulk of the papilloma [132]. A similar strategy has also demonstrated the cell non-autonomous recruitment of untargeted cells in skin cancer models driven by the carcinogens 7,12-dimethylbenz(a)anthracene (DMBA) and 2-*O*-tetradecanoylphorbol-13-acetate (TPA) [133, 134]. Finally, the expression of oncogenic β-catenin in K19 + and Lgr5 + stem cells of the hair follicle has been shown to induce outgrowths and benign tumours which are mostly composed of untargeted cells as shown by lineage tracing [135–137]. Therefore, plenty of evidence exists supporting the notion that paracrine signalling can initiate tumour formation and fuel progression.

### Evidence for senescence-induced tumorigenesis

There is ample in vitro and in vivo evidence demonstrating the tumour promoting activities of cellular senescence on cells carrying oncogenic mutations (see reviews cited above). On the other hand, the requirement of senescence and SASP signalling for the cell non-autonomous *initiation* of tumours (as it occurs in mouse ACP models) has been less explored, possibly due to the inherent difficulty in studying the early stages of cell transformation and tumour initiation in vivo.

Although a considerable number of in vitro experiments have shown that conditioned media from senescent cell cultures can enhance the cancerous properties of preneoplastic or neoplastic cells (see the reviews mentioned above), it has also been shown that some cells require exposure to senescent cell-conditioned media to acquire cancer stem cell-like phenotypes and the ability to form tumours when transplanted into nude mice [138–140]. In vivo, senescence and the SASP-driven paracrine tumourigenesis have been convincingly demonstrated to occur in several tissues of *D. melanogaster*. In this model, elegant use of gene targeting, genetic lineage tracing and cell ablation approaches have shown that activating oncogenic Ras induces senescence-like phenotypes and SASP activation in targeted cells, which lead to tumour growth in neighbouring wild type cells through the activation of developmental pathways and JNK signalling [141–144]. In a zebrafish model of melanoma, researchers showed that telomere shortening promotes the accumulation of senescent cells which produces an inflammatory environment that significantly fosters the formation of melanomas [145]. This, taken together with the fact that senescent cells accumulate during aging in several tissues and that their elimination in mice causes a significant delay in cancer deaths [146–149], implicates senescent cells in age-related tumourigenesis.

In mice, the injection of senescent HRasV12-expressing keratinocytes can induce the formation of skin papillomas that are very similar to those in DMBA/TPA models. Strikingly, a considerable portion of cells within the papillomas contain non-targeted host cells, suggesting an important role for senescence-driven paracrine signalling in tumour formation [150]. Notably, a recent study revealed that p16^INK4A^-expressing senescent keratinocytes and their SASP induce hyperproliferation and activation of WNT signalling target genes in neighbouring naïve cells, leading to the formation of papillomas when DMBA/TPA is applied [151]. These findings are remarkably similar to the previously discussed studies involving the paracrine recruitment of untargeted cells in skin tumours, thus raising interesting questions about the contributions of senescence and the SASP in those models [132–137].

There is also strong evidence suggesting that senescence and the SASP can drive paracrine tumourigenesis in the liver. For example, it has been shown that obese mice develop higher levels of the microbial metabolite deoxycholic acid (DCA) which leads to higher number of senescent hepatic stellate cells (HSCs) and increased SASP expression. Interestingly, depletion of either the senescent HSCs or knockout of IL1B signalling (a common SASP component) significantly prevents the growth of DMBA-induced hepatocellular carcinomas [152]. Moreover, other studies have shown the requirement of senescent fibroblasts and their SASP for the induction of liver tumours from transplanted cancer cell lines [39, 153]. In particular, it has been shown that the pro-tumourigenic effects of the SASP can be inhibited by knockdown of PTBP-1, a factor controlling the alternative splicing of genes involved in intracellular trafficking [39]. The tumour-initiating role of senescence has further been supported by a recent study showing that carcinogen-induced liver tumour formation requires the SASP from senescent HSCs and that it can be prevented by chemically ablating senescent cells [154].

The evidence discussed above suggests three conceivable mechanisms, acting alone or in combination, that could mediate senescence-mediated paracrine tumourigenesis: (1) induction of proliferation in neighbouring cells, therefore, promoting genomic instability and the appearance of novel mutations [155, 156]; (2) promotion of cell reprogramming and plasticity, which has already been shown to occur in the contexts of cancer, aging and repair during injury both in vivo and in vitro [21, 150, 157–159]; (3) activation of developmental pathways (such as WNT signalling) related to the induction, maintenance and survival of cancer stem cell phenotypes [160, 161]. Our findings from both humans and mouse models suggest all 3 mechanisms are involved in ACP pathogenesis. First, proliferating cells are often found in close proximity to the senescent clusters, while the expression of the stem cell factor SOX9 is increased in these neighbouring cells. Importantly, the presence of both proliferating and SOX9 + cells is diminished in the context of an attenuated SASP [42, 75]. Second, mouse ACP pituitaries contain more and faster growing pituitary progenitors/stem cells as determined by colony-forming assays [75]. Finally, the senescent clusters in both mouse and human possess a transcriptional profile that is analogous to the enamel knot, while their SASP contains many developmental signalling factors from the WNT, SHH, BMP and FGF families that are critical for tooth morphogenesis [67].

## New therapeutic opportunities: senolytics and SASP-modulating drugs

The discovery that senescent cells, through the SASP, are determinant factors of age-related pathogenesis and drivers of organismal ageing has led to the identification and development of drugs able to counteract their damaging effects. Senotherapies aim mostly to either kill senescent cells selectively, by targeting key survival critical pathways, or to regulate their paracrine activities using SASP-modulating drugs. In a couple of milestone studies, Baker and colleagues have demonstrated that the genetic ablation of p16-expressing senescent cells is able to delay ageing-associated disorders [146, 162]. Subsequently, three independent studies have shown that senescent cells can specifically be killed using certain senolytic compounds, resulting in the rejuvenation of tissue stem cells [163–165]. These initial studies have been followed by more investigations targeting senescent cells in different disease contexts that have together provided solid evidence for a critical contribution of senescence in several age-related diseases [166–170]. Senolytic therapies have thoroughly been reviewed elsewhere [27, 171, 172]

The previously discussed cell autonomous and non-autonomous functions of senescent cells in tumourigenesis have provided a strong rationale to explore the potential of senotherapies to prevent or delay tumour formation. Senotherapies could improve anticancer treatments, as many currently used therapies may lead to the induction and accumulation of senescent cells in the tumour bed, which could be detrimental for the patients and result in recurrence. In support of this, it has been shown that genetic or chemical ablation of senescent cells that are induced after anti-cancer therapy significantly reduces tumour recurrence in breast and liver cancer models [25, 173].

In ACP models, initial experiments have shown that the genetic attenuation of the senescence/SASP response is able to reduce significantly the tumour-inducing potential of the senescent cell clusters [42]. In human ACP, the inhibition of IL6 (a SASP factor expressed in mouse and human ACP) results in reduced motility of ACP epithelial tumour cells, suggesting that SASP attenuation may reduce tumour burden in vivo [174]. A number of studies have shown that senescent cluster cells in the embryonic mouse ACP model can be ablated chemically using senolytic agents in vitro. Treatment of pre-tumoural mouse pituitaries with ABT-263 and ABT-737, two well-studied senolytics that target the anti-apoptotic proteins Bcl2, Bcl-xL and Bcl-w [164, 165], results in smaller clusters due to cell apoptosis in ex vivo explants cultures [42, 175]. Likewise, ouabain and digoxin, two members of the cardiac glycoside family of organic compounds, can induce apoptosis in senescent cluster cells [175]. In a recent report, a galactose-modified cytotoxic prodrug has been used to preferentially kill the cluster senescent cells in vitro based on the higher levels of β-galactosidase in these cells [176]. Therefore, mouse ACP models can potentially be used to study novel senolytic compounds in the context of senescence-mediated paracrine tumourigenesis in vivo. Preclinical studies are still required to establish proof-of-principle evidence that senotherapies may be relevant against craniopharyngioma and therefore mouse ACP models are ideal tools to test these novel treatments.

## Conclusions

Early studies showing a negative correlation between senescent cell burden and tumour progression towards malignancy helped establish cellular senescence as a tumour-suppressive mechanism. However, when viewed under the precepts of widely accepted cell autonomous paradigms of tumorigenesis, this inverse senescence-cancer progression relationship implies that cells carrying oncogenic driver mutations have to either bypass or escape the senescent phenotype for tumours to progress. Although there are some convincing experiments in vitro [161, 177], conclusive evidence demonstrating the occurrence of either senescence bypass or escape has yet to be produced in vivo [178]. Of relevance, a genome-wide methylation analysis has shown that the methylation signature of transformed cells is acquired stochastically and independently of the senescent epigenetic state, which argues against the senescence escape hypothesis [179]. Likewise, although *NOTCH1 is* the most commonly mutated gene in the physiologically aged human oesophagus (followed by *TP53*), *NOTCH1* mutations are considerably underrepresented in oesophageal cancers, suggesting these are more likely to evolve from epithelial cells without *NOTCH1* mutations. In contrast, *TP53* mutations, which are several-fold less frequent in the aged oesophagus, are almost universally present in oesophageal cancers, suggesting that these cancers originate from the small fraction of *TP53* mutant cells [180, 181]. These findings also argue against senescence escape as an underlying mechanism of oesophageal cancer development, as it would be expected for mutations in *NOTCH1* to be as abundant as *TP53*. More recently, the mechanisms of action of known carcinogens have been challenged. By analysing the mutational signatures of tumours generated in mice exposed to one of 20 carcinogens, it has been found that most of these agents are not directly mutagenic on the genome (i.e. they do not increase mutation burden in the tumours), with most mutations, including driver mutations, resulting from tissue-specific endogenous processes. This suggests that these carcinogens promote tumour initiation in a different manner, possibly by creating a permissive environment that allows tumour growth rather than, as initially believed, by increasing mutation rate [182]. Therefore, a combination of techniques such as genetic lineage-tracing, transplantation and sequencing strategies will be required to demonstrate that oncogene-targeted cells can escape or avoid the senescent phenotype in animal models of cancer.

The ACP models have revealed that the relationship between oncogenic driver-mutations, tumour cells-of-origin and the tumour microenvironment (TME) can be much more complex than what can be explained by conventional models of carcinogenesis. Lineage tracing has shown the otherwise counterintuitive observation that tumours can be formed from cells that are not related to the original oncogenic insult. Molecular studies in these models indicate that senescent cells bring about a pro-tumourigenic TME that induces tumour initiation in a paracrine manner through the SASP, while in human ACP, the evidence strongly supports a role of senescent cells in tumour growth and invasion. Further preclinical research using senolytics in the ACP mouse models may pave the way to clinical trials to evaluate the clinical relevance of senotherapies, alone or in combination, against human ACP.

It remains to be seen how common the occurrence of senescence-induced paracrine tumourigenesis in other tumours and cancers is, but it will be particularly interesting to assess the role of senescent cells and the SASP in the cancer models for which cell non-autonomous initiating mechanisms have already been described (Sect. 4.1 and Table 1). Evidence of the prevalence of such mechanisms in the origin or establishment of some human cancers will also have to be produced, which may be difficult due to the fact that early stages of tumourigenesis are hard to get hold of in humans. However, within the context of therapy-induced senescence, it may be easier to demonstrate the pro-tumourigenic effects of both senescent tumoural cells and senescent cells within the TME. Therefore, therapies capable of inducing senescent phenotypes such as radiotherapy, chemotherapy or even targeted approaches, in combination with senolytics or SASP-modulators [183], could significantly improve the current standard of care and result in better clinical outcomes.
